# Hybridization of an invasive shrub affects tolerance and resistance to defoliation by a biological control agent

**DOI:** 10.1111/eva.12134

**Published:** 2014-01-15

**Authors:** Wyatt I Williams, Jonathan M Friedman, John F Gaskin, Andrew P Norton

**Affiliations:** 1 Department of Bioagricultural Sciences and Pest Management, Colorado State UniversityFort Collins, CO, USA; 2 Oregon Department of Forestry, Private Forests DivisionSalem, OR, USA; 3 U.S. Geological Survey, Fort Collins Science CenterFort Collins, CO, USA; 4 USDA, Agricultural Research ServiceSidney, MT, USA

**Keywords:** biological control, hybridization, latitudinal clines, rapid evolution, resistance, *Tamarix* sp., tolerance

## Abstract

Evolution has contributed to the successful invasion of exotic plant species in their introduced ranges, but how evolution affects particular control strategies is still under evaluation. For instance, classical biological control, a common strategy involving the utilization of highly specific natural enemies to control exotic pests, may be negatively affected by host hybridization because of shifts in plant traits, such as root allocation or chemical constituents. We investigated introgression between two parent species of the invasive shrub tamarisk (*Tamarix* spp.) in the western United States, and how differences in plant traits affect interactions with a biological control agent. Introgression varied strongly with latitude of origin and was highly correlated with plant performance. Increased levels of *T. ramosissima* introgression resulted in both higher investment in roots and tolerance to defoliation and less resistance to insect attack. Because tamarisk hybridization occurs predictably on the western U.S. landscape, managers may be able to exploit this information to maximize control efforts. Genetic differentiation in plant traits in this system underpins the importance of plant hybridization and may explain why some biological control releases are more successful than others.

## Introduction

Rapid evolution is an important process contributing to the success of some invasive plant species in their introduced ranges (for reviews, see Lee 2002; Bossdorf et al. 2005; Keller and Taylor 2008; Prentis et al. 2008). Some introduced plant species have evolved faster growth, higher fecundity, or altered allocation of herbivore defenses compared to populations in the native ranges (Blossey and Notzold 1995; Hull-Sanders et al. 2007). Moreover, evolution is invoked to explain why populations of several exotic plant species persist at low numbers for decades before undergoing exponential population growth (e.g., termed ‘evolution of invasiveness’, Ellstrand and Schierenbeck 2000). Invasive species provide a unique opportunity to study evolution in contemporary time; it is also imperative to understand the evolutionary dynamics that influence invasion success because these organisms are related to declines in biodiversity, drastic changes in ecosystem function, and costs of over $120 billion annually in the United States alone (Wilcove et al. 1998; Mack et al. 2000; Pimentel et al. 2005).

Evolutionary mechanisms, such as founder events, intra-and interspecific hybridization, and adaptation, have all resulted in significant evolutionary change in the introduced ranges (Lee 2002; Bossdorf et al. 2005; Prentis et al. 2008). For instance, despite several deleterious effects, hybridization can also result in novel genotypes which are better suited to their environment than either parent species (Lee 2002; Donovan et al. 2010). Hybridization has been implicated in the transfer of beneficial genes for traits such as cold hardiness or resistance to fungal diseases and herbivores (Snow et al. 1999; Abbott et al. 2003; Whitney et al. 2006; Rieseberg et al. 2007). Selection can act on these novel hybrids, spreading beneficial alleles rapidly throughout populations of invasive plants (Keller and Taylor 2008). Indeed, hybridization has been implicated in numerous plant invasions (Ellstrand and Schierenbeck 2000; Schierenbeck and Ellstrand 2009), with several hybrid taxa evolving larger size or higher fecundity than either parent species or outcompeting and replacing parent species (Campbell et al. 2006; Whitney et al. 2006; Ridley and Ellstrand 2010). Invasive plant hybrids indirectly threaten native communities and pose difficulty to land managers who are responsible for their control (Vila et al. 2000; Blair et al. 2008).

One effective tool for managing invasive plants is classical biological control, where specialized natural enemies are imported and released to provide top-down effects on their hosts (DeBach 1964; van Klinken and Raghu 2006). How biological control is affected by invasive plant hybridization and other evolutionary mechanisms remains largely unknown (Muller-Scharer et al. 2004). In native systems, herbivores can distinguish among hybrid genotypes (Fritz et al. 1998; McGuire and Johnson 2006), and hybrid plants can have more or less resistance to attack compared with their parent species (Whitham 1989; Fritz et al. 1999; Krebs et al. 2011). In invasive plant systems, hybridization can affect the frequency of herbivore attack, especially when hybrids are compared as a group to their parent species (Blair et al. 2008; Krebs et al. 2011; Cuda et al. 2012). However, little is known about how susceptibility to herbivory varies across levels of species introgression in invasive plants. This information could be useful in cases where invasions are comprised almost entirely of hybrid genotypes (Williams et al. 2007; Gaskin and Kazmer 2009). Clearly, more research is needed to determine the extent to which invasive plant hybridization affects the efficacy of classical biological control.

In addition to hybridization, another mechanism of evolution important to invasive plants is adaptation to abiotic stress (Bossdorf et al. 2005; Ridley and Ellstrand 2010). Introduced plants are exposed to novel climatic and edaphic conditions in the new range on which selection can act. One way this selection is evident is through the evolution of latitudinal clines. When grown in a common environment, populations of several exotic plant species exhibit inherited genetic differences in traits, such as size, phenology, and cold hardiness, reflecting the climate from the latitude where they were collected (Weber and Schmid 1998; Kollmann and Bañuelos 2004; Maron et al. 2004; Leger and Rice 2007; Montague et al. 2008; Keller et al. 2009). While latitudinal clines of native plant species have been well documented for quite some time (Turreson 1930; Clausen et al. 1940), similar patterns are also seen for non-native plants, some of which have evolutionary histories as little as 100 years in their new ranges (Ridley and Ellstrand 2010; Hodgins and Rieseberg 2011). It is unclear whether evolution of strategies to cope with abiotic stress may interact with biological control. For instance, common garden experiments by Friedman et al. (2008, 2011) show that northern populations of *Tamarix* spp. have evolved increased cold hardiness due to extreme minimum temperatures and have likely adapted to winter dieback by allocating more resources to belowground tissues (e.g., coarse roots). In turn, we hypothesize that these same individuals may have increased tolerance to leaf herbivory because they have more carbohydrate reserves available for leaf flush following defoliation. In short, evolution of life history traits to cope with abiotic stress could also influence ecological interactions with specialized natural enemies, consequently affecting the success of biological control efforts.

Here, we investigate the effects of hybridization on plant traits and latitudinal clines of the perennial shrub tamarisk (i.e., saltcedar, *Tamarix* spp., family Tamaricaceae). Several species of tamarisk were introduced to the United States in the nineteenth century to stabilize stream banks and to serve as ornamental plants (Robinson 1965). By the mid-1900s, at least four species were considered serious pests in the arid west (Gaskin and Schaal 2002; Gaskin et al. 2012). Tamarisk has been estimated to occupy at least 360 000 ha in the western United States (Nagler et al. 2011) and is the second most dominant woody riparian species (in terms of percent cover) in the western United States (Friedman et al. 2005). In 2001, the biological control agent *Diorhabda carinulata* Brullé (Coleoptera: Chrysomelidae) was released at field sites across western North America. In some locations, populations of this insect became well established and control of tamarisk is being achieved, while at other sites, the control agent has failed to establish despite repeated attempts (DeLoach et al. 2003, 2009). Some authors have speculated that genetic makeup of host plants may play a role in these instances (Gaskin and Schaal 2002; Gaskin and Kazmer 2009; Dudley et al. 2012; Hultine et al. 2013). Could differences in susceptibility among hybrid *Tamarix* genotypes be driving the success or failure of tamarisk biological control?

A hybrid ‘swarm’ of F1, F2, and backcrosses to two parent species, *T. ramosissima* and *T. chinensis*, make up the bulk of the tamarisk invasion in the western United States (Friedman et al. 2008; Gaskin and Kazmer 2009). Based on diagnostic markers and AFLP data, Gaskin and Kazmer (2009) estimated 83–87% of genotypes collected across several sites in western North America were indeed *T. ramosissima* x *chinensis* hybrids. Interestingly, despite repeated sampling, only a few hybrid individuals have been found in the native range (Gaskin, pers. comm.). Therefore, hybridization appears to be a major part of the tamarisk invasion in the United States, but it is unknown exactly how hybridization influences defense against herbivory (Gaskin and Kazmer 2009; Moran et al. 2009). In a set of experiments investigating cold hardiness and leaf phenology of hybrid tamarisk populations, Friedman et al. (2008, 2011) demonstrated inherited latitudinal variation in these traits. When plants were grown in a common environment, southern populations had significantly increased susceptibility to cold temperatures and later onset of fall leaf senescence compared to northern populations. There is no indication to what degree this latitudinal variation results from species introgression or whether variation in these traits influences interactions with biological control (e.g., tolerance and regrowth following defoliation).

We used a DNA fingerprinting technique to quantify species introgression among tamarisk plants collected from a wide geographical range in the western United States. Then, we compared introgression values against several environmental conditions at the collection sites for their power in predicting tamarisk performance traits when plants were grown in a common garden and subsequently subjected to defoliation experiments. Information on genetic differences in tamarisk growth and defense, along with knowledge of hybrid plant distribution, can potentially improve biological control in this system where evolution for at least some plant traits (i.e., cold hardiness, leaf phenology) has occurred. For instance, if tamarisk hybridization occurs in a predictable pattern across the western United States and certain tamarisk hybrids have evolved more or less susceptibility to feeding by *D. carinulata*, this information can be applied to improve the efficiency of biological control tactics.

## Materials and methods

### Plant material

A large common garden consisting of nearly 350 tamarisk shrubs located in Fort Collins, Colorado, was the source of plant material for this study (see Friedman et al. 2008, 2011). All plants were originally collected from 15 natural populations along a latitudinal gradient from Montana to Texas where *D. carinulata* had yet to establish. Plants were 3 years old at the onset of the current study. The large garden excluded vertebrate herbivores, and *D. carinulata* was never observed at the site. For the experiments described below, 72 genotypes representing 14 populations were randomly sampled from the large garden (Table S1). Six to ten cuttings (25 cm long, 2–8 mm diameter) were harvested from each of the selected genotypes. The cuttings were dipped in rooting hormone, planted individually in 100% perlite, and placed on a mist bench for 4 weeks to stimulate root growth. Survivors were transplanted into 13 (W) × 13 (W) × 30 (H) cm pots with a mixture of 80:20 of potting soil and sand. These plants were kept in the greenhouse under constant temperature and light for several weeks and then transferred to an outdoor shade house on the campus of Colorado State University, Fort Collins, Colorado.

### AFLPs

For DNA fingerprinting, we used clones of all available genotypes from the large common garden (*n* = 324; Friedman et al. 2008) plus data available from other North American plants (*n* = 47) as well as native *T. ramosissima* (*n* = 56) and *T. chinensis* (*n* = 65) collected in Asia (Gaskin and Kazmer 2009). Genomic DNA was extracted from approximately 20 mg of silica-dried plant material using a modified CTAB method (Hillis et al. 1996). Fragment PCR and AFLP analysis used the same protocol and platform found in Gaskin and Kazmer (2009). In short, primer pairs used for amplification were MseI + CTA/EcoRI + ACC and MseI + CTA/EcoRI + ACT, and fragments were analyzed using an Applied Biosystems 3130 Genetic Analyzer (Foster City, CA, USA). GeneMapper v4.0 (Applied Biosystems) was used to visualize fragments for presence and length, while Structure v2.3.3 (Pritchard et al. 2000; Falush et al. 2003; Hubisz et al. 2009) was used to calculate assignment scores and introgression levels in terms of one of the two parent species, *T. ramosissima* (hereafter referred to as ‘introgression’). One can calculate *T. chinensis* introgression by simple subtraction (100-%*T. ramosissima* introgression). Plants were considered hybrids if introgression levels were <0.9 or >0.1 and posterior probability intervals did not reach 1.0 (Pritchard et al. 2000; Blair and Hufbauer 2010).

### Performance and tolerance experiment

Ten populations representing a large geographical area were selected for the plant performance and tolerance experiment (Table S1). From these populations, 43 genotypes had at least three clones that survived the propagation technique outlined above. The three most vigorous clones per genotype were randomly assigned to one defoliation treatment: herbivore, chemical, and control. Finally, after clones were assigned treatments, they were further randomly assigned to experimental blocks housed in 100 (W) × 100 (W) × 30 (H) cm wood box frames, which aided in treatment prescriptions and accounted for spatial heterogeneity in the outdoor shade house. There were nine total blocks (three per treatment).

For the herbivore treatment, on July 16, 2010, we introduced 150 adult *D. carinulata* (number chosen based on our experience with the insect at field sites) into each of three blocks assigned to the herbivore defoliation treatment. The beetles were collected from an established field population near Palisade, Colorado, and were starved 24 h to minimize any effects of host preference based on prior feeding experience. To contain them within the blocks, we placed cages constructed from lightweight polyester cloth and plastic tubing over the box frames. We removed these cages after 14 days when adults reached the end of their life cycle and larvae had not yet hatched from egg masses. Once emerged, larvae were free to move and feed among overlapping plant canopies within each block, while canopies from neighboring blocks were kept isolated. Larvae, which are not highly mobile, were never recorded outside of the blocks to which they were assigned. Blocks of the other defoliation treatments (chemical and control) were caged in the same manner and timing as those in the herbivore treatment.

Because we anticipated that some clones in the herbivore treatment would receive little or no defoliation, we included a chemical treatment to measure plant response to nearly complete defoliation. For plants in the three blocks assigned to chemical defoliation treatment, the nonsystemic, defoliating herbicide, carfentrazone-ethyl (Aim™, FMC Corp. Philadelphia, PA, USA) was applied with a four-nozzle boom and a backpack sprayer using two passes at a volume of 187 L per hectare, which is consistent with field application of this herbicide (S. Nissen, pers. comm.). We temporarily moved the plants 200 m outside of the garden for approximately 2 h to avoid spray drift to nontarget plants. The timing of the application purposefully coincided with the height of beetle defoliation (August 13, 2010). The remaining three blocks were assigned to the control treatment, and plants contained in these blocks did not receive any prescribed defoliation.

From June to October 2010, measurements of damage and plant performance were recorded (see below). During this period, plants were watered every third day and supplemented with nutrients monthly using 20-20-20 NPK fertilizer. From October 2010 to April 2011, the wood boxes were filled with mulch to prevent roots from freezing, and water was supplemented as needed. In April 2011, the plants were uncovered, and the watering and fertilizing regime continued until the end of the experiment in June 2011 when all plants were harvested.

### Quantifying damage, plant performance, and tolerance

We used percentage of plant canopy damaged as a measure of defoliation. On September 3, 2010, we took two digital photographs (both in horizontal perspective) of each plant against a black background. The photographs were analyzed using ImageJ, which quantifies pixilated area of digital images based on color spectrum (v1.44, U.S. National Institute of Health, http://imagej.nih.gov/ij/). The whole canopy area was determined by setting the hue threshold from 21–102, while the green canopy area was measured with a hue threshold from 47–102. We averaged the whole and green canopy areas for pairs of pictures for each plant. By comparing the green canopy to the whole canopy area, we were able to assess the percentage of canopy damaged for each plant, which included yellow and brown desiccated portions of plant canopy characteristic of *D. carinulata* damage. Our technique of quantifying defoliation matched closely to a subjective score of percent plant damage (*r* = 0.94, *P* < 0.0001).

Annual canopy growth was used as a proxy for plant fitness, necessary for calculating tolerance to defoliation. We used canopy growth in lieu of reproductive output given the age of tree clones and the duration of this study. In June 2010 (before defoliation treatments were applied) and again in June 2011, plant height and the perpendicular canopy widths were used to calculate canopy volume. This measure of canopy volume was strongly correlated with canopy area calculated using digital images and ImageJ software (*r* = 0.79, *P* < 0.0001). Canopy area was log-transformed in order to meet model assumptions of anova, and percent annual growth was calculated using these transformed scores of canopy size.

We followed the suggestion of Strauss and Agrawal (1999) when determining plant tolerance to defoliation. Tolerance was quantified as the slope of the relationship between canopy damage and fitness measurement at 1 year postdefoliation treatment for each of the 43 genotypes. A separate slope was calculated for each genotype using three pairs of damage and associated fitness scores (i.e., herbivore, chemical, and control replicates). A positive slope indicates overcompensation for defoliation, while a negative slope reveals undercompensation.

To detect biomass allocation differences 1 year after defoliation treatments, we harvested all plants at the end of the experiment (June 2011). The roots were carefully washed of potting soil and placed in separate paper bags for drying. Green foliage and woody aboveground growth (excluding the original cutting) were also put in separate bags. The samples were placed in an oven at 55°C until they were dry (72 h). We used a digital balance to record the dry mass of green foliage, woody stems, coarse roots, and fine roots. Coarse roots were defined as those ≥1 mm diameter and fine roots as those <1 mm in diameter. Root-to-shoot ratios were calculated by dividing total belowground biomass by total aboveground biomass. Cutting diameter only increased minimally over the course of the experiment and was not used to investigate the effects of defoliation or other factors. However, we included initial cutting diameter as covariate in models investigating overall plant performance (see below).

### Resistance experiment

We developed a second experiment to investigate resistance of tamarisk genotypes to herbivory by *D. carinulata* because of the difficulty in accurately measuring feeding damage by this particular herbivore. Four populations from the large garden were chosen based upon their dispersed geographical origin with 7–8 genotypes per population and up to eight clones per genotype surviving the propagation technique (Table S1). In August 2010, after these cuttings were transplanted and established in the outdoor garden for 10 weeks, we clipped ∼30 g of fresh green foliage from each plant, placed the material in a paper sack, and dried for 3 days at 40°C. Then, we powdered each sample using a coffee grinder, cleaning the instrument with 70% EtOH and drying between samples. Next, we added 5 g of dried plant material, 750 mg of agar, and 30 ml of H_2_O to individual, flat-bottomed glass test tubes. After mixing the solution, the test tubes were placed in a steam bath for 20 min and then cooled to room temperature.

Although our procedure likely destroyed or altered plant volatiles, it allowed us to produce uniform feeding media representing each individual clone grown in the garden. This media were used in an initial bioassay – the first of its kind for *Diorhabda* and *Tamarix* – to determine whether there were feeding differences among hybrid plants. Two identical pellets media were extracted from the tubes using a 5-mm punch. A single pellet was placed on top of filter paper inside of a 50-mm plastic Petri dish. One dish was randomly chosen as a control (no larvae), while the other received five, preweighed second-instar *D. carinulata* larvae collected from the field site in Palisade, Colorado. The larvae were starved for 24 h before the experiment began to minimize the effects of prior feeding experience. The Petri dishes were placed in an environmental chamber for 48 h with a daily temperature (29°C/21°C) and light cycle (14-h L/8-h D). After 48 h, larvae were removed and weighed. The pellets were then dried at 45°C for 3 day. The dry masses of the ‘herbivore’ and ‘control’ pellets were then compared to obtain percent consumed. Thus, we obtained both a resistance score and associated larval mass for several clones of each plant genotype. We developed this pellet bioassay in response to the difficulty in measuring actual damage to tamarisk plants by *D. carinulata* feeding (i.e., abscission of whole stems regardless of amount of feeding damage; difficulty of quantifying feeding damage on three-dimensional photosynthetic stems). Moreover, fresh-picked tamarisk shoots dry quickly (<1 h) and are not consumed by *D. carinulata* (pers. obs.); thus, our approach circumnavigated issues with using live plant material.

### Statistical analysis

We used Akaike's information criterion (AIC) as a means of model selection to determine whether introgression sufficiently explained variation in plant traits compared to abiotic factors from the population origins (Burnham and Anderson 2002). We used PROC MIXED (SAS v9.3, SAS Institute, Cary, NC, USA) with maximum-likelihood estimation of error to calculate AICc scores. We also calculated Akaike weights (wi) and evidence ratios, both of which are used as additional techniques for determining the most informative model (Burnham and Anderson 2002). Five model sets were considered for the model selection (Table S2). One model focused on species introgression, while the other four models investigated the effects of local adaptation to latitude (proxy of daylength), average number of annual frost-free days, average annual minimum temperature, and elevation of sites where tamarisk was collected. To characterize temperature variation at the collection sites, we used the DAYMET interpolated daily low temperature data for the period 1980–2003 gridded at 1-km resolution (Thornton et al. 1997, http://www.daymet.org).

After using model selection to determine whether hybridization or adaptation to abiotic conditions at site origins was more important in predicting plant growth traits, we then employed mixed-model analysis of covariance (ancova) for the investigation of defoliation damage and plant performance measurements using the statistical program JMP v9.0.2 (SAS Institute, Cary NC, USA). Defoliation treatment and experimental block were considered fixed effects (i.e., both factors were tested across all possible levels), while plant subject was modeled as a random effect (i.e., levels sampled from a larger population). Diameter of stem cuttings at the onset of plant propagation was used as a covariate to control for the effect of initial size. In the resistance experiment, variables in models included the random effect of plant subject and either introgression or one of the four environmental variables. For tolerance, we used a weighted regression analysis to determine the strength of the relationship between species introgression and tolerance to defoliation. The weights were calculated as 1/*σ*^2^ from each of the 43 tolerance regressions (damage vs. fitness for each genotype). Finally, we used Student's *t*-tests (*α *= 0.05) for *post hoc* comparisons among group means of treatment groups.

## Results

### AFLPs and environmental conditions at tamarisk sites

**Figure 1:**
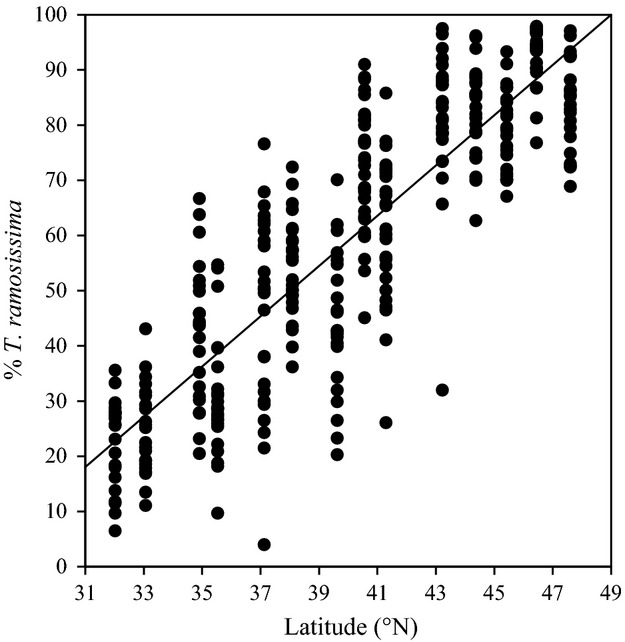
The relationship between tamarisk species introgression, expressed as percentage *T. ramosissima*, and latitude of population origin (%*T. ramosissima* introgression = 4.6*latitude − 123; *R*^2 ^= 0.74; *n* = 342). See Table S1 for detailed site descriptions.

Tamarisk introgression was highly correlated with latitude of plant origin (*r* = 0.87), annual frost-free days (*r* = 0.78), and minimum temperatures (*r* = 0.87; all *P *< 0.0001) and moderately correlated with elevation (*r* = 0.41; *P* < 0.01) at sites where plant material was collected. The environmental conditions were correlated with each other with the exception of elevation, which was only moderately correlated with annual frost-free days (*r* = 0.59, *P* = 0.03) and not correlated with latitude or minimum temperatures. The amount of tamarisk introgression increased with increasing latitude in the western United States (%*T. ramosissima* introgression = 4.6*latitude − 123; anova:*F*_1,340_ = 987, *P* < 0.0001; Fig. 1, Table S1). Tamarisk from Colorado River, Texas (32.0^o^N), had the lowest amount of *T. ramosissima* introgression (22.7 ± 1.7%), while tamarisk from Musselshell River, Montana (46.4^o^N), had the highest amount of *T. ramosissima* introgression (92.9 ± 1.1%). Mean introgression across all latitudes was 59.3 ± 1.4%. Out of the 342 U.S. tamarisk shrubs included in the AFLP analysis, there were 37 individuals with assignment scores >0.9 for *T. ramosissima* (all from sites 41.3–47.6^o^N) and only four individuals with assignment scores >0.9 for *T. chinensis* (all from 32.0–35.5 ^o^N).

### Model selection

Because introgression was highly correlated with latitude, frost-free days, and minimum temperatures of population origin, these predictor variables could not be placed in the same model investigating plant performance, thus justifying the use model selection. AIC scores showed that Model 1 (introgression) was more informative than the other models for root-to-shoot ratio, resistance, and larval mass (Table S3). For plant biomass, introgression and latitude were essentially equal in their ability to fit the observed data (Δ_*i*_ AIC < 2). Only in one case (canopy growth rate) was an environmental variable, elevation, more informative than introgression. All models predicting defoliation damage had Δ_*i*_ AIC < 2, indicating no clear distinction among these candidates (Burnham and Anderson 2002). In summary, introgression explained as much or more variation in plant response to defoliation than abiotic factors from population origins; thus, we present the reminder of the results in terms of tamarisk introgression.

### Plant performance traits

We provide full ancova reports for all measured plant responses in Appendix S1. Initial plant size (cutting diameter) had a significant influence on all aspects of plant biomass, while it had no effect on defoliation damage, growth rate, or root-to-shoot ratio. The random effect of plant subject explained 20–60% of variation across all plant traits.

The introgression*treatment interaction was not significant for defoliation damage, indicating no difference in damage across introgression levels, nor was there a significant effect of introgression alone on this response variable (Appendix S1). However, there was a significant effect of treatment type on plant damage (*F*_2,77.6_ = 120, *P* < 0.0001). Damage from the chemical treatment (LS mean ± SE: 63.9 ± 2.6%, range: 20–93%) was greater than damage from the herbivore treatment (LS mean ± SE: 35.1 ± 2.6%, range: 3–94%). Both chemical and herbivore treatments resulted in significantly greater plant damage than the control treatment (LS mean ± SE: 7.5 ± 2.6%, range: 1–32%).

Our proxy for plant fitness, annual canopy growth rate, was also not affected by species introgression nor was there a significant interaction between introgression and defoliation (Appendix S1). Defoliation treatment had a significant effect on growth rate (*F*_2,77.6_ = 120, *P* < 0.0001). Growth rates between the treatments were 37.7 ± 5.0%, 33.3 ± 5.0%, and 11.63 ± 6.1%, for control, herbivore, and chemical defoliation, respectively. Fitness scores ranged between −100.0 and 93.6% annual canopy growth. Six of the 43 clones assigned to the chemical treatment died during the winter following the treatment application (fitness = −100% canopy growth). These plants were retained in the analysis of tolerance to defoliation (see below).

**Figure 2:**
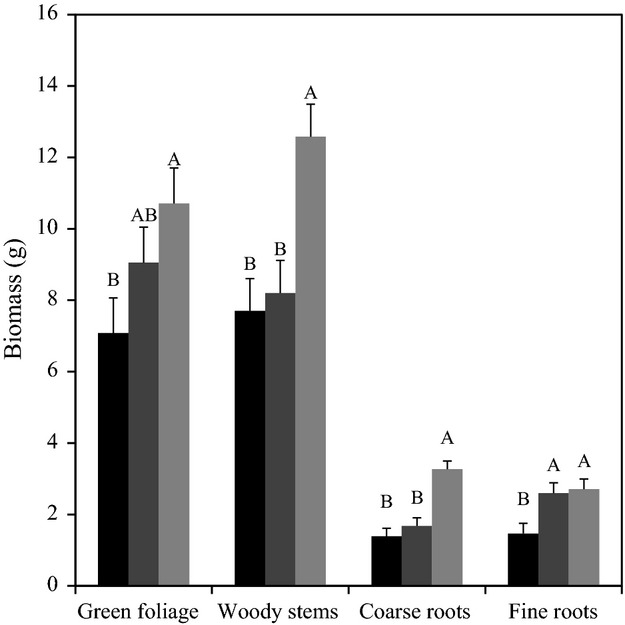
LS mean biomass ± SE of four plant tissue groups for three defoliation treatments. Chemical: black bars; herbivore: dark gray bars; control: light gray bars. Different letters correspond to differences between means within a plant tissue category (Student's *t*-test, *α *= 0.05). Defoliation treatments were applied in the summer of 2010, and plant parts were harvested, dried, and weighed the following spring. Mean percent canopy defoliation was 64%, 35%, and 7.5% for chemical, herbivore, and control treatments, respectively.

Total plant biomass decreased with species introgression (biomass = 21.0–0.17*introgression; *F*_1,39.7 _= 4.7, *P* = 0.04). In addition, defoliation treatment had a significant effect on plant biomass (*F*_2, 74.5 _= 16.6, *P* < 0.0001), but the interaction between treatment and introgression was not significant. Defoliation by either chemical or herbivore treatments led to significantly reduced coarse root and woody stem biomass (Fig. 2). On the other hand, only plants that were chemically defoliated had significantly lower green foliage and fine root biomass compared to control plants.

Results of ancova exposed both a significant treatment effect (*F*_2,78.6 _= 8.5, *P* < 0.001) and a significant effect of species introgression (*F*_1,40.5_ = 4.5, *P *= 0.04) on root-to-shoot ratio, but not a significant interaction between these two factors. Plants in the herbivore (LS mean: 0.29 ± 0.02) and control (LS mean: 0.27 ± 0.02) treatment groups both had significantly greater root-to-shoot ratios than plants in the chemical treatment (LS mean: 0.19 ± 0.02), but were not significantly different than each other. Introgression showed a positive relationship with root-to-shoot ratios (ratio = 0.00116*introgression + 0.20), implying that plants with high levels of *T. ramosissima* introgression invest more in belowground rather than in aboveground growth.

### Tolerance

**Figure 3:**
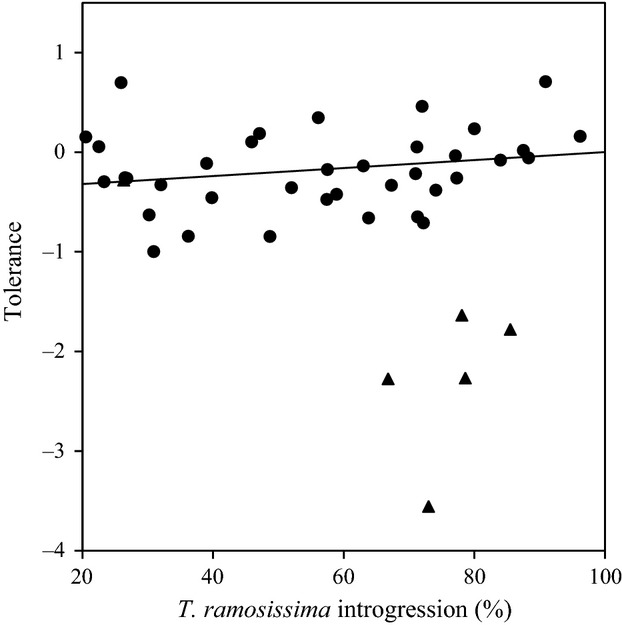
Tolerance to defoliation across species introgression for plant genotypes in the experiment. Positive tolerance values correspond to plant overcompensation, while negative values correspond to plant undercompensation to defoliation. Tolerance demonstrated a positive relationship with introgression when the SEs of tolerance scores were included in a weighted regression (tolerance = 0.004*introgression – 0.40; *R*^2^ = 0.13). Weighted regression was used because some clones in the chemical treatment died, thus leading to more error for those particular genotypes (triangles). See Appendix S2 for detailed tolerance scores of each genotype.

We provide a summary of tolerance slopes and standard errors for each of the 43 genotypes in Appendix S2. These genotypes, which were haphazardly chosen from the larger pool of 342 plants, had a mean species introgression level of 58.0 ± 3.4% *T. ramosissima* (range: 20.5–96.2%). Tolerance demonstrated a positive relationship with introgression using weighted regression analysis (tolerance = 0.004*introgression – 0.40; *F*_1,41_ = 6.27, *P* = 0.02). Plants with high levels of *T. ramosissima* introgression had increased tolerance to defoliation (Fig. 3).

### Resistance

**Figure 4:**
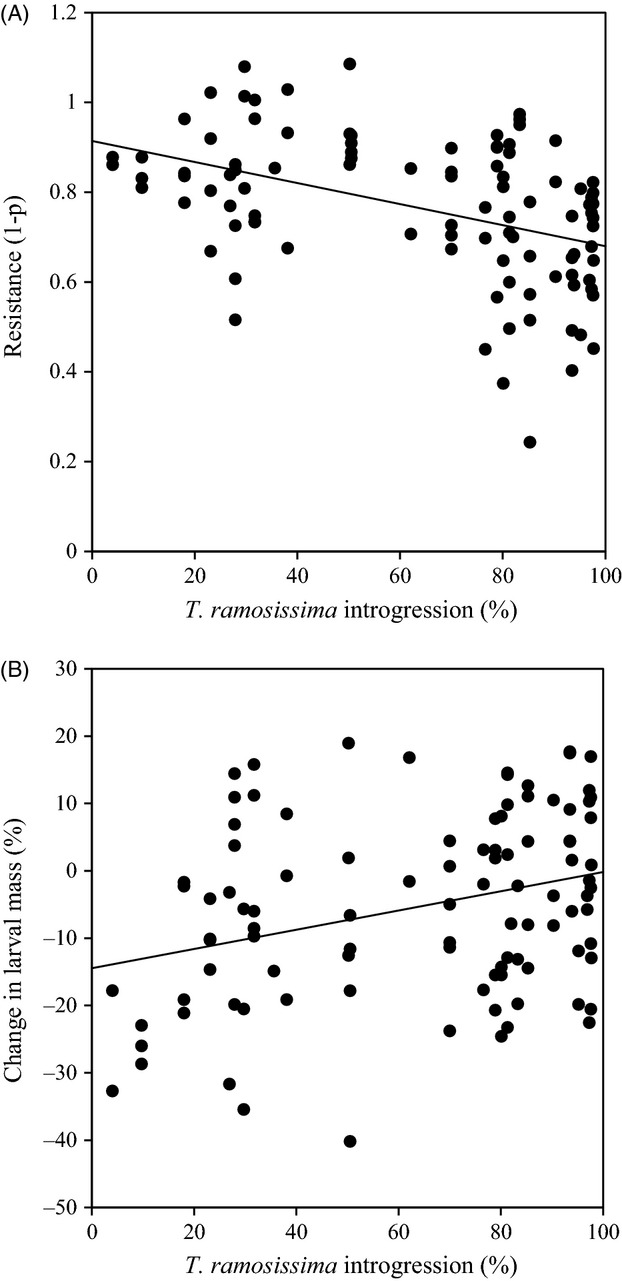
Plant genotype resistance to herbivory by *D. carinulata* in the pellet bioassay (A). Resistance is defined as 1-proportion of pellet damage. Pellets made from plants with high levels of *T. ramosissima* introgression had greater resistance (resistance = 0.919–0.002*introgression; *F*_1,28.9_ = 15.2, *P* < 0.001; *R*^2^ = 0.42). Some resistance values were >1 in some cases because the treatment pellet weighed slightly more than the control pellet and it was not consumed. Percent change in larvae mass in the pellet bioassay (B). Plant species introgression had a negative effect on larval performance (%change in larval mass = 0.15*introgression – 15.2; *F*_1,27.8 _= 8.0, *P* < 0.01; *R*^2^ = 0.29).

High resistance to herbivory was reflected by a small proportion, p, of plant material consumed in the pellet bioassay. There was a negative relationship between species introgression and resistance (*F*_1,28.9 _= 15.2, *P* < 0.001; Fig. 4A): Pellets with high levels of *T. ramosissima* were more completely consumed. Additionally, larvae used in this experiment gained more mass when fed pellets with high *T. ramosissima* introgression (*F*_1,28.9 _= 8.0, *P* < 0.01; Fig. 4B), confirming the low resistance of these plant genotypes.

## Discussion

Tamarisk evolution via hybridization is widespread in North America. Using markers for a single-locus nuclear DNA gene (*PepC*), Gaskin and Schaal (2002) first reported that the most common plant in the U.S. invasion was a hybrid of two species-specific genotypes that were geographically isolated in the native range of Asia. Then, as follow-up, Gaskin and Kazmer (2009) used a multilocus approach across all genomic DNA (i.e., AFLP) and Bayesian cluster analysis to determine that 83–87% of the U.S. tamarisk genotypes are indeed hybrids between *T. ramosissima* and *T. chinensis* parent species. In the present study, which used the same AFLP protocol and laboratory as Gaskin and Kazmer (2009), we found that 89% of the 371 North American plants in the study were hybrids with only handful of individuals (10% and 1%, respectively) classified as *T. ramosissima* and *T. chinensis* parent species. Additionally, we found a significant relationship between latitude of tamarisk populations and interspecific hybridization, following similar studies (Friedman et al. 2008; Gaskin and Kazmer 2009); however, we describe the pattern in detail for the first time: Among tamarisk populations in the western United States, every one degree increase in latitude between 32.0 and 46.7^o^N corresponds to a 4.6% positive change in *T. ramosissima* introgression (Fig. 1). As our experiments revealed, the relationship between latitude and tamarisk hybridization has direct impact on interactions with a specialized herbivore introduced for control of this invasive plant.

The exact cause of the latitudinal cline in tamarisk hybridization is unknown. One possible mechanism is that hybridization occurred in nurseries in the early 1800s before tamarisk was introduced at multiple locations in the western United States (Gaskin et al. 2012), while another is that several independent hybridization events could have occurred in areas where both parent species were co-introduced (Gaskin and Kazmer 2009; Friedman et al. 2011). Under either scenario, subsequent sorting out of adapted hybrid genotypes then occurred postintroduction. The abundance of tamarisk hybrids in North America compared to the lack of interspecific hybridization in the native range in Asia suggests that species introgression has played an important role for invasion success (Gaskin and Kazmer 2009; Gaskin et al. 2012). Similar patterns of latitude and genetic variation are demonstrated by at least one other invasive shrub, *Eupatorium adenophorum* (Huang et al. 2009).

We used Akaike's information criterion (AIC) in a model selection approach to determine the relative strengths of the correlated factors of introgression and abiotic conditions at population origins (i.e., hybridization and local adaptation) in explaining plant growth and defense traits (Burnham and Anderson 2002). Introgression was more informative than other models in many cases, including those for tissue allocation and resistance (Table S3). On the other hand, growth rate was strongly related to elevation, which most likely resulted from the unimodal relationship between elevation and latitude of collection sites. For total biomass, introgression and latitude explained equal amounts of variation and both had a significant negative relationship with this plant trait (Table S3). Latitudinal clines in similar traits have been recorded in a number of invasive plant species in their introduced ranges (Weber and Schmid 1998; Kollmann and Bañuelos 2004; Maron et al. 2004; Leger and Rice 2007; Montague et al. 2008; Keller et al. 2009). We propose that in the case of tamarisk latitudinal clines, interspecific hybridization may have provided the sufficient genetic material for selection to act upon following introduction to North America. Regardless of the tight correlation between latitude and introgression, genetic information (e.g., AFLPs), and not simply geographical location, was more informative for root-to-shoot ratios and herbivore defense strategies for hybrid tamarisk plants.

In the outdoor garden experiment, there were a significant decrease in total plant biomass and a significant increase in root-to-shoot ratios in plants grown with increasing percentage of *T. ramosissima* alleles. Sexton et al. (2002) observed the variation in root-to-shoot ratios of comparable tamarisk populations, with northern populations investing more in belowground growth. Additionally, we found that plants with high levels of *T. ramosissima* introgression were more tolerant of defoliation than plants with low levels of *T. ramosissima* introgression (Fig. 3). Given that belowground carbohydrate stores are important for tamarisk leaf flush following defoliation (Hudgeons et al. 2007), tamarisk hybridization is important in an ecological and evolutionary sense. For instance, plants from northern populations may experience more dieback as the result of extreme low temperatures, selecting for genotypes with greater belowground allocation and root-to-shoot ratios (Friedman et al. 2008). Indeed, *T. ramosissima* introgression was associated with higher latitudes and higher root-to-shoot ratios in our experiment. *T. ramosissima* genotypes may have adapted strategies to cope with *D. carinulata* outbreaks in the native range where they co-occur (Lewis et al. 2003), while in the introduced range, *D. carinulata* has caused the most impact on tamarisk populations whose introgression values are <60% *T. ramosissima* (DeLoach et al. 2009; Gaskin and Kazmer 2009). We have begun investigating levels of introgression at field sites where *D. carinulata* has failed to establish to determine whether this pattern holds true. In addition, at least five species of *Diorhabda* co-occur with numerous *Tamarix* spp. in the native range of Asia and Europe, but no *Diorhabda* species has been recorded on *T. chinensis* in eastern Asia (Tracy and Robbins 2009). Thus, *T. chinensis*-dominated genotypes may invest more in chemical defenses against herbivores in general than to allocating resources to regrowth following aboveground dieback. Given that tolerance is an herbivore defense strategy that has a strong degree of inheritance (Strauss and Agrawal 1999; Agrawal et al. 2004), hybridization between *T. ramosissima* and *T. chinensis* could affect the outcome of biological control at certain field sites.

In the resistance experiment, the overall trend in larval mass indicated that, in fact, most insects lost weight during the 48-h experiment, indicating that plant pellets would be a poor choice for an artificial diet. Still, differences in weight gained (or lost) were evident among plant genotypes, and these differences coincided with tamarisk introgression. Plant pellets with high levels of *T. ramosissima* introgression were consumed in higher quantities than their counterparts (Fig. 4A). Similarly, larvae that were fed pellets made from plants with high *T. ramosissima* introgression performed better (Fig. 4B). Several accessions and species of tamarisk have been tested in *D. carinulata* feeding trials, oftentimes with significant differences in attack or insect development (DeLoach et al. 2003; Lewis et al. 2003; Milbrath and DeLoach 2006; Dalin et al. 2009; Moran et al. 2009). We did not detect differences in resistance among genotypes for the outdoor study, even though we used a precise digital imaging tool to objectively assess damage. One explanation is that when tissue is damaged by *D. carinulata* feeding, a common plant response is to abscise entire leaves and stems. Thus, beetles in the outdoor experiment could have preferentially fed upon various tamarisk hybrids, but the amount of herbivory was masked by this abscission. Nonetheless, the bioassay experiment confirmed differences in resistance among hybrid genotypes, and these differences were related to tamarisk introgression.

Differences in herbivore resistance among hybrid plant genotypes occur in natural systems (Fritz et al. 1998; McGuire and Johnson 2006; Krebs et al. 2011). A possible mechanism for these differences is novel chemistry of hybrid host plants (Orians 2000). Three reviews involving over 80 plant taxa show that 5.5% of secondary compounds of hybrid genotypes are novel compared to either parent species, while nearly 20% of hybrid plants either over-or underexpress secondary compounds compared to their parents. Moreover, roughly 5–6% of hybrid genotypes have higher resistance to herbivory compared to parent species (Orians 2000; Cheng et al. 2011). Because herbivorous insects use plant chemistry as a means of host recognition, novel chemical combinations due to hybridization may make plants unrecognizable or unpalatable to highly specialized herbivores (Schoonhoven et al. 2005). While plant-based chemical attractants are known for *D. carinulata* (Cossé et al. 2006), there is no information whether these chemicals differ among hybrid genotypes. Furthermore, there are no published records of tamarisk chemistry relative to insect herbivory in the introduced range. It would be interesting to see whether differences in tamarisk resistance to herbivory can be explained by differences in the production of important secondary metabolites by hybrid tamarisk plants.

Finally, our study also revealed an interesting ecological relationship between defoliation and plant performance, which may have implications in an applied sense. Only a modest amount of damage by beetles (mean = 35.1%) significantly reduced annual growth of woody stems and coarse roots compared to control plants (mean damage = 7.5%, most likely attributed to grasshoppers observed in the outdoor shade house). Moreover, beetle defoliation had the same effect as did a larger amount of damage (mean = 63.9%) by chemical defoliation (Fig. 2). Therefore, *D. carinulata* appears to reduce allocation to these structural and storage tissues as much as chemical defoliation. On the other hand, chemical defoliation by carfentrazone-ethyl significantly reduced the growth of photosynthetic tissue and fine roots the following year, while defoliation by herbivores did not. While chemical control appears to have a more immediate effect in terms of overall plant size, our experiment did not take into account that plants often experience repeated herbivore defoliation in areas where the insect is established. At field sites where *D. carinulata* has had the most impact, the insect completes 2–4 generations per year while depleting carbohydrate reserves through repeated defoliation, ultimately leading to plant mortality in as little as 3 years (Lewis et al. 2003; Hudgeons et al. 2007; Pattison et al. 2011).

Adaptation appears to be an important evolutionary mechanism for hybrid tamarisk populations in terms of particular life history traits (e.g., cold hardiness and leaf phenology; Friedman et al. 2008, 2011) and tolerance and resistance to a specialized herbivore (this study). The source of genetic variation for selection to act upon in this system may have been due to interspecific hybridization. Quantitative trait loci mapping and breeding experiments involving parent species to produce F1, F2, and backcrossed offspring are necessary to be definitive (Cheng et al. 2011). Additionally, comparing parent species from the native range to parental and hybrid genotypes from the introduced range would reveal how important hybridization is in this system (Keller and Taylor 2008; Ridley and Ellstrand 2010). In terms of assessing the strength of latitudinal clines, caution should be taken when drawing inferences from a single common garden experiment (Williams et al. 2008; Colautti et al. 2009). Multiple common gardens with plants collected from a wide range of latitudes in both ranges are ideal for determining whether evolution of latitudinal clines has occurred (Maron et al. 2004; Colautti et al. 2009). Given that a study which combines both latitude and hybridization may be difficult, perhaps focusing attention on U.S. populations that naturally possess a wide range of genetic variability could be fruitful.

Rapid evolution can affect outcomes in the application of biological control (see July 2012 special issue of Evolutionary Applications). Curiously, the tamarisk system has been an example of two such cases, both involving the biology of the insect (Thomas et al. 2010; Bean et al. 2012). Here, we show that evolution of tamarisk via hybridization affects interactions between plants and a specialized herbivore introduced for its control. High tolerance of northern tamarisk (i.e., *T. ramosissima*-dominated genotypes) is apparently related to belowground tissue allocation, while high herbivore resistance of southern tamarisk (i.e., *T. chinensis*-dominated genotypes) may be due to chemical or structural defenses. Intense herbivory by *D. carinulata* may cause rapid natural selection of hybrid plants combining tolerance and resistance more successfully than either parent species alone. In the short term, if hybrids are spread across the landscape in a predictable manner, as is the case with *Tamarix* invasion in the western United States, this knowledge regarding the response of particular hybrids to herbivory can be exploited to maximize biological control efforts.

## Data archiving statement

Data for this study are available from the Dryad Digital Repository: http://dx.doi.org/10.5061/dryad.vc62h.

## Supporting Information

Appendix S1 ANCOVA results for models investigating the effects of tamarisk introgression on various plant traits.

Appendix S2 Tolerance scores and associated test statistics for each of the 43 plant genotypes involved in the outdoor garden study.

Table S1 Site information, including latitude, elevation, frost-free days, annual extreme minimum temperature, and average species introgression levels for each of the tamarisk populations in the current study.

Table S2 Competing models used in the Akaike's information criterion (AIC) model selection.

Table S3 Results of Akaike's information criterion (AIC) model selection.
