# Supplementary material for: Hybridization of an invasive shrub affects tolerance and resistance to defoliation by a biological control agent
Source: Evol Appl. 2014 Jan 15;7(3):381–93. doi: 10.1111/eva.12134 (PMC3962298; doi:10.1111/eva.12134)
Supplement: Appendix S1 — ANCOVA results for models investigating the effects of tamarisk introgression on various plant traits. [file eva0007-0381-sd1.docx]

Appendix S1. ANCOVA results for the effects of tamarisk introgression on various plant traits. Response variables included plant damage from the defoliation treatments, canopy growth rate (estimate of plant fitness), biomass at the end of the experiment, and the ratio of belowground to aboveground growth for plants in the outdoor garden experiment. Pellet damage and larval mass refer to the plant genotype resistance and larval performance, respectively, in the pellet bioassay experiment. Plant variation refers to the amount of variation explained by the random variable in the model, plant subject.

| Response variable | R^2^ | Treatment *F*(df) | Introgr. *F*(df) | T x I *F*(df) | Block *F*(df) | Initial size *F*(df) | Plant (%) |
| --- | --- | --- | --- | --- | --- | --- | --- |
| Defoliation damage | 0.71 | 121.3(2,77.8)*** | 0.23(1,39.2) | 0.39(2,78.3) | 3.1(1,115)** | 1.9(1,83.5) | 2.5 |
| Canopy growth rate | 0.43 | 9.4(2,74.5)*** | 0.06(1,36.7) | 0.40(2,74.6) | 2.0(6,109) | 0.77(1,97.5) | 17.3 |
| Total biomass | 0.78 | 17.0(2,75.2)*** | 4.7(1,39.7)* | 2.2(2,74.7) | 1.3(6,89.7) | 22.2(1,113)*** | 55.3 |
| Aboveground | 0.77 | 13.9(2,75.3)*** | 4.9(1,39.8)* | 2.5(2,74.8)† | 1.6(6,89.9) | 19.7(1,113.1)*** | 55.2 |
| Green foliage | 0.70 | 6.7(2,76.0)** | 1.3(1,40.2) | 2.2(2,75.5) | 1.7(6,92.9) | 7.3(1,115)** | 49.4 |
| Woody stems | 0.82 | 21.2(2,74.7)*** | 8.8(1,39.4)** | 4.3(2,74.2)* | 1.1(6,87.5) | 32.2(1,111)*** | 59.7 |
| Belowground | 0.72 | 22.4(2,75.0)*** | 3.3(1,40.0)† | 1.0(2,74.5) | 0.67(6,94.5) | 22.7(1,115)*** | 44.6 |
| Fine roots | 0.68 | 11.8(2,76.7)*** | 1.7(1,40.4) | 1.4(2,76.2) | 1.4(6,97.3) | 19.9(1,115)*** | 41.0 |
| Coarse roots | 0.73 | 32.9(2,73.7)*** | 4.0(1,37.6)† | 0.52(2,73.2) | 0.52(6,93.7) | 15.2(1,115)*** | 44.4 |
| Roots:Shoots | 0.45 | 8.5(2,78.6)*** | 4.5(1,40.5)* | 1.2(1,78.4) | 5.3(6,112)*** | 0.40(1,95.5) | 10.5 |
| Pellet resistance | 0.42 | NA | 15.2(1,28.9)*** | NA | NA | NA | 19.4 |
| Larval mass | 0.29 | NA | 8.0(1,27.8)** | NA | NA | NA | 14.3 |

***, *P* < 0.001; **, *P* < 0.01; *, *P* < 0.05; †, *P* < 0.1
