# Supplementary material for: Hybridization of an invasive shrub affects tolerance and resistance to defoliation by a biological control agent
Source: Evol Appl. 2014 Jan 15;7(3):381–93. doi: 10.1111/eva.12134 (PMC3962298; doi:10.1111/eva.12134)
Supplement: Appendix S2 — Tolerance scores and associated test statistics for each of the 43 plant genotypes involved in the outdoor garden study. [file eva0007-0381-sd2.docx]

Appendix S2. Tolerance scores for each of the 43 genotypes in the outdoor garden study. Tolerance refers to the linear relationship between percent canopy defoliation and fitness. A negative slope shows under-compensation for defoliation while a positive slope indicates over-compensation. All P values <0.1 (slope different from zero) are shown in bold. The latitude of origin and species introgression for each plant subject is provided. *, indicates that the clones for these particular genotypes assigned to the chemical treatment died.

| Genotype | Latitude | Introgression | Tolerance | SE | t ratio | P value |
| --- | --- | --- | --- | --- | --- | --- |
| 421 | 33.063 | 36.2 | -0.843 | 0.103 | -8.17 | **0.08** |
| 556 | 33.063 | 22.5 | 0.056 | 0.307 | 0.18 | 0.89 |
| 602* | 33.063 | 26.3 | -0.281 | 1.917 | -0.15 | 0.91 |
| 122 | 34.904 | 39.0 | -0.113 | 0.241 | -0.47 | 0.72 |
| 187 | 34.904 | 30.9 | -0.997 | 0.417 | -2.39 | 0.25 |
| 349 | 34.904 | 45.9 | 0.103 | 2.450 | 0.04 | 0.97 |
| 463 | 34.904 | 20.5 | 0.153 | 0.126 | 1.22 | 0.44 |
| 471 | 34.904 | 30.2 | -0.630 | 0.100 | -6.27 | 0.10 |
| 591 | 34.904 | 63.8 | -0.660 | 0.499 | -1.32 | 0.41 |
| 481 | 35.529 | 25.9 | 0.699 | 0.992 | 0.70 | 0.61 |
| 514 | 35.529 | 26.5 | -0.256 | 0.214 | -1.20 | 0.44 |
| 561 | 35.529 | 26.8 | -0.262 | 0.290 | -0.90 | 0.53 |
| 86 | 38.087 | 52.0 | -0.356 | 0.046 | -7.76 | **0.08** |
| 302 | 38.087 | 57.5 | -0.174 | 0.136 | -1.28 | 0.42 |
| 411 | 38.087 | 57.4 | -0.474 | 0.198 | -2.39 | 0.25 |
| 488 | 38.087 | 56.1 | 0.347 | 0.443 | 0.78 | 0.58 |
| 502 | 38.087 | 58.9 | -0.422 | 0.229 | -1.84 | 0.32 |
| 644 | 38.087 | 39.8 | -0.457 | 0.225 | -2.03 | 0.29 |
| 41 | 39.623 | 23.3 | -0.296 | 0.007 | -44.23 | **0.01** |
| 636 | 39.623 | 32.0 | -0.327 | 0.039 | -8.42 | **0.08** |
| 671 | 39.623 | 48.7 | -0.846 | 0.100 | -8.42 | **0.08** |
| 107 | 40.559 | 77.3 | -0.260 | 0.303 | -0.86 | 0.55 |
| 110* | 40.559 | 66.8 | -2.274 | 0.102 | -22.38 | **0.03** |
| 166 | 40.559 | 63.0 | -0.137 | 0.173 | -0.79 | 0.57 |
| 243 | 40.559 | 88.3 | -0.057 | 0.055 | -1.03 | 0.49 |
| 261 | 40.559 | 71.0 | -0.215 | 0.260 | -0.83 | 0.56 |
| 352 | 40.559 | 74.1 | -0.381 | 0.046 | -8.28 | **0.08** |
| 649 | 40.559 | 80.0 | 0.235 | 0.290 | 0.81 | 0.57 |
| 46 | 41.291 | 72.2 | -0.710 | 0.283 | -2.51 | 0.24 |
| 139 | 41.291 | 71.3 | -0.649 | 0.047 | -13.67 | **0.05** |
| 149 | 41.291 | 77.1 | -0.036 | 0.217 | -0.16 | 0.90 |
| 645 | 41.291 | 47.1 | 0.187 | 0.107 | 1.76 | 0.33 |
| 672 | 41.291 | 67.3 | -0.332 | 1.208 | -0.27 | 0.83 |
| 548 | 43.222 | 90.9 | 0.708 | 0.096 | 7.37 | **0.09** |
| 583 | 43.222 | 84.0 | -0.080 | 0.026 | -3.10 | 0.20 |
| 643* | 43.222 | 78.6 | -2.267 | 0.530 | -4.28 | 0.15 |
| 30 | 45.427 | 87.5 | 0.019 | 0.285 | 0.07 | 0.96 |
| 231 | 45.427 | 72.0 | 0.460 | 0.030 | 15.18 | **0.04** |
| 270* | 45.427 | 78.1 | -1.636 | 1.764 | -0.93 | 0.52 |
| 449 | 45.427 | 71.2 | 0.053 | 0.123 | 0.43 | 0.74 |
| 239 | 47.604 | 96.2 | 0.160 | 0.148 | 1.08 | 0.48 |
| 240* | 47.604 | 85.5 | -1.778 | 0.423 | -4.21 | 0.15 |
| 244* | 47.604 | 73.0 | -3.555 | 0.773 | -4.60 | 0.14 |
