# Supplementary material for: Hybridization of an invasive shrub affects tolerance and resistance to defoliation by a biological control agent
Source: Evol Appl. 2014 Jan 15;7(3):381–93. doi: 10.1111/eva.12134 (PMC3962298; doi:10.1111/eva.12134)
Supplement: Table S1 — Site information, including latitude, elevation, frost-free days, annual extreme minimum temperature, and average species introgression levels for each of the tamarisk populations in the current study. [file eva0007-0381-sd3.docx]

Table S1. Mean species introgression with standard error calculated as percentage *T. ramosissima* based on AFLP genetic fingerprinting and subsequent assignment analysis of 342 total plants. Latitude, longitude, elevation, average frost free days, and average annual minimum temperature of sites where Friedman et al. (2008) made original collections. N1, number of plant genotypes used in AFLP analysis; N2 and r, number of genotypes and replicate clones, respectively, used in defoliation experiments; *, Populations used in the plant performance and tolerance experiment; †, Populations used in the resistance experiment; ‡, Location of the outdoor common garden.

| Population | Avg | SE | Lat(^o^N) | Lng(^o^W) | El(m) | FFD | MinT | N1 | N2 | r |
| --- | --- | --- | --- | --- | --- | --- | --- | --- | --- | --- |
| Colorado River, TX^†^ | 22.7 | 1.7 | 32.020 | -100.737 | 579 | 306.9 | -10.9 | 23 | 7 | 22 |
| Lake Alan Henry, TX* | 24.7 | 1.7 | 33.063 | -101.042 | 648 | 295.2 | -12.3 | 22 | 3 | 9 |
| Buffalo Lake, TX* | 42.3 | 2.7 | 34.904 | -102.118 | 1106 | 248.8 | -16.6 | 22 | 6 | 18 |
| Lake Meredith, TX* | 30.6 | 2.2 | 35.529 | -101.767 | 897 | 256.3 | -16.5 | 24 | 3 | 9 |
| Cimarron River, OK^†^ | 46.2 | 3.5 | 37.122 | -101.892 | 1031 | 226.4 | -20.6 | 25 | 7 | 22 |
| Arkansas River, CO* | 54.2 | 1.8 | 38.087 | -102.288 | 1056 | 211.1 | -22.2 | 25 | 6 | 18 |
| Bonny Reservoir, CO* | 44.1 | 2.8 | 39.623 | -102.194 | 1121 | 202.5 | -23.9 | 22 | 3 | 9 |
| Poudre River, CO* | 71.9 | 2.0 | 40.559 | -105.015 | 1489 | 200.9 | -24.6 | 32 | 7 | 21 |
| Lake McConaughy, NE* | 60.9 | 2.7 | 41.291 | -101.933 | 999 | 197.5 | -26.4 | 25 | 5 | 15 |
| Boysen Reservoir, WY* | 82.1 | 2.5 | 43.222 | -108.180 | 1443 | 182.9 | -28.7 | 26 | 3 | 9 |
| Keyhole Reservoir, WY^†^ | 82.4 | 1.7 | 44.368 | -104.792 | 1251 | 177.3 | -30.6 | 25 | 7 | 30 |
| Powder River, MT* | 78.9 | 1.5 | 45.427 | -105.405 | 923 | 184.2 | -31.8 | 24 | 4 | 12 |
| Musselshell River, MT^†^ | 92.9 | 1.1 | 46.445 | -108.525 | 967 | 195.7 | -30.2 | 25 | 8 | 26 |
| Fort Peck Reservoir, MT* | 83.8 | 1.8 | 47.604 | -106.902 | 686 | 184.6 | -33.8 | 22 | 3 | 9 |
| Fort Collins, CO^‡^ | - | - | 40.573 | -105.082 | 1529 | - | - | - | - | - |
