# Supplementary material for: Hybridization of an invasive shrub affects tolerance and resistance to defoliation by a biological control agent
Source: Evol Appl. 2014 Jan 15;7(3):381–93. doi: 10.1111/eva.12134 (PMC3962298; doi:10.1111/eva.12134)
Supplement: Table S2 — Competing models used in the Akaike's information criterion (AIC) model selection. [file eva0007-0381-sd4.docx]

Table S2. Competing models used in AIC model selection. Model 1 investigates the influence of *Tamarix* species introgression while models 2-5 focus on the importance of environmental variables from population origins on tamarisk performance and herbivore defense. Introgression and environmental factors were never combined in candidate models because of the high correlation among these predictor variables. *Only these factors were included in models investigating resistance in the bioassay experiment.

| Factors in model | Model 1 | Model 2 | Model 3 | Model 4 | Model 5 |
| --- | --- | --- | --- | --- | --- |
| Defoliation treatment | X | X | X | X | X |
| Introgression* | X |  |  |  |  |
| Treatment x Introgression | X |  |  |  |  |
| Latitude* |  | X |  |  |  |
| Treatment x Latitude |  | X |  |  |  |
| Frost free days* |  |  | X |  |  |
| Treatment x Frost free days |  |  | X |  |  |
| Annual minimum temperature* |  |  |  | X |  |
| Treatment x Min. temperature |  |  |  | X |  |
| Elevation* |  |  |  |  | X |
| Treatment x Elevation |  |  |  |  | X |
| Block | X | X | X | X | X |
| Plant subject* | X | X | X | X | X |
| Initial plant size | X | X | X | X | X |
