# Supplementary material for: Hybridization of an invasive shrub affects tolerance and resistance to defoliation by a biological control agent
Source: Evol Appl. 2014 Jan 15;7(3):381–93. doi: 10.1111/eva.12134 (PMC3962298; doi:10.1111/eva.12134)
Supplement: Table S3 — Results of Akaike's information criterion (AIC) model selection. [file eva0007-0381-sd5.docx]

Table S3. AIC results of competing models for six response variables. Because the ratios of observations to the number of parameters in each model <40, AIC scores were converted to AICc to account for small sample sizes. A lower AICc score for a given response variable indicates that more variation is explained using that particular model than competitor models. Differences in AICc scores (∆*_i_*) between a competing model and the best fit model is an indication of model selection. A ∆*_i_*, < 2 indicates no clear best model, 4<∆*_i_*, <7 indicates less evidence for a competing model, while ∆*_i_*, >10 means there is essentially no support for the competing model. Akaike weights (*w_i_*) are used to calculate evidence ratios. An evidence ratio of three means that there is relatively little evidence for that model.

| Dependent variable | Model | AICc | ∆*_i_* | *w_i_* | Evid. ratio |
| --- | --- | --- | --- | --- | --- |
| Defoliation damage | 1 | 1114.6 | 0 | 0.25 | 1.0 |
|  | 2 | 1115.3 | 0.7 | 0.18 | 1.4 |
|  | 3 | 1115.1 | 0.5 | 0.19 | 1.3 |
|  | 4 | 1115.3 | 0.7 | 0.18 | 1.4 |
|  | 5 | 1115.0 | 0.4 | 0.20 | 1.2 |
|  |  |  |  |  |  |
| Canopy growth | 1 | 1283.4 | 8.1 | 0.01 | 57 |
|  | 2 | 1279.4 | 4.1 | 0.10 | 7.8 |
|  | 3 | 1281.6 | 6.3 | 0.03 | 23 |
|  | 4 | 1280.4 | 5.1 | 0.06 | 13 |
|  | 5 | 1275.3 | 0 | 0.79 | 1.0 |
|  |  |  |  |  |  |
| Biomass | 1 | 1018.3 | 0 | 0.50 | 1.0 |
|  | 2 | 1019.4 | 1.1 | 0.29 | 1.7 |
|  | 3 | 1024.3 | 6.0 | 0.02 | 20 |
|  | 4 | 1020.3 | 2.0 | 0.18 | 2.7 |
|  | 5 | 1026.5 | 8.2 | 0.01 | 60 |
|  |  |  |  |  |  |
| Root:shoot | 1 | -154.0 | 0 | 0.73 | 1.0 |
|  | 2 | -149.4 | 4.6 | 0.07 | 10 |
|  | 3 | -148.8 | 5.2 | 0.05 | 13 |
|  | 4 | -149.1 | 4.9 | 0.06 | 12 |
|  | 5 | -149.5 | 4.5 | 0.08 | 9.5 |
|  |  |  |  |  |  |
| Pellet resistance | 1 | 824.6 | 0 | 0.76 | 1.0 |
|  | 2 | 827.7 | 3.1 | 0.16 | 4.7 |
|  | 3 | 832.5 | 7.9 | 0.01 | 52 |
|  | 4 | 829.8 | 5.2 | 0.06 | 13 |
|  | 5 | 836.2 | 11.7 | 0.00 | 347 |
|  |  |  |  |  |  |
| Larval mass | 1 | 801.6 | 0 | 0.54 | 1.0 |
|  | 2 | 804.0 | 2.4 | 0.16 | 3.3 |
|  | 3 | 804.7 | 3.1 | 0.11 | 4.7 |
|  | 4 | 804.3 | 2.7 | 0.14 | 3.9 |
|  | 5 | 806.7 | 5.1 | 0.04 | 13 |
